# Mycotic Pseudoaneurysm: Clinical Course, Management and Prognosis

**DOI:** 10.7759/cureus.28408

**Published:** 2022-08-25

**Authors:** Moe Ameri, Julian Gonzalez-fraga, Joseph Orndorff, Alexandra E Ecker, Annie Cherner, Kirav P Patel

**Affiliations:** 1 Internal Medicine, University of Texas Medical Branch at Galveston, Galveston, USA; 2 Internal Medicine, University of Texas Medical Branch at Galveston, Galveston , USA; 3 Internal Medicine, UTMB John Sealy School of Medicine, Galveston, USA; 4 Internal Medicine, UTMB John Sealy School of Medicine, Galveston , USA

**Keywords:** prolonged hospitalization, generic antibiotics, mycotic pseudoaneurysm, infectious disease pathology, infectious disease, general internal medicine

## Abstract

Pseudoaneurysm of vessels (most common in arteries), in general, happens when a blood vessel wall is damaged leading to leakage of blood and its collection in the surrounding tissue, essentially resulting in a false aneurysm. These false collections can be problematic and can develop in any location. However, their most common clinical presentation is in the femoral arteries. These manifest especially following an endovascular intervention. Here, we discuss a case of a 73-year-old male whose in-hospital course was complicated by the development and subsequent infection of a pseudoaneurysm after he was admitted for sepsis from a UTI. We further highlight the pathophysiology related to the formation of a pseudoaneurysm, and the mechanism of action behind various treatment modalities used. The clinical course and possible treatment options may vary. However, a robust combination of early surgical management alongside medical management seems to provide the best outcome.

## Introduction

Pseudoaneurysms occur when a compromise in the arterial wall leads to the dissection of blood into the surrounding tissue, creating a sac that surrounds the artery and is continuous with its lumen. This sac's formation is either limited to one of the two outer layers (tunica adventitia and tunica media) of the artery, or within the soft tissue surrounding the vessel, or within the soft tissue surrounding the artery. The annual incidence of mycotic pseudoaneurysm of the common femoral artery ranges from 0.03% to 1% The most common clinical presentation of a pseudoaneurysm is a femoral pseudoaneurysm; less common sites where they may present alternatively are visceral pseudoaneurysms and aortic pseudoaneurysms [1]. Common symptoms of pseudoaneurysm are due to swelling and can include pain and nerve compression in severe cases [2]. Other possible complications can be rupture, deep vein thrombosis, or infection. Mycotic aneurysms are the result of a superimposed infection and most often occur in the femoral artery or abdominal aorta. Like other superinfections, this can be due to bacteremia, local injury with inoculation, spread from neighboring tissues, or septic emboli [3-7]. Typically, the causative organisms involved include *Staphylococcus spp, Salmonella spp*, and *Pseudomonas aeruginosa* [8,9]. Rarely, other organisms, such as Streptococcus and Klebsiella, are involved [10]. Here, we describe a rare case of a mycotic pseudoaneurysm caused by *Proteus mirabilis*, a pathogen commonly affecting the kidneys and causing struvite renal calculi.

## Case presentation

The patient is a 73-year-old male who initially presented with a complicated urinary tract infection, gram-negative bacteremia complicated by sepsis, encephalopathy, and acute kidney injury. He had a complex past medical history which included rheumatic fever as a child, paroxysmal atrial fibrillation, heart failure with preserved ejection fraction, and left internal carotid stenosis. Moreover, his past surgical history included multiple stenting & bypass procedures for his coronary artery disease and mechanical thrombectomy following two cerebral vascular accidents, depicting a significant arterial burden. Recent history included a diagnostic left heart catheterization via right femoral artery access and watchman closure via right femoral vein access for the history of remote GI bleed while on warfarin. The patient was lost to follow-up after these procedures, but he presented approximately six months later with acute exacerbation of heart failure and cardiorenal syndrome. His condition improved with diuresis, but a right lower extremity duplex ultrasound obtained for leg pain showed an 11 x 5.5 cm (patent area 7.4 x 3 cm) pseudoaneurysm. A Vascular Surgery consult was obtained at that time. No surgical intervention was recommended given the chronicity and lack of symptoms as his leg pain had improved and was likely unrelated to the pseudoaneurysm. The patient was discharged with a plan for outpatient follow-up with vascular surgery.

**Figure 1:**
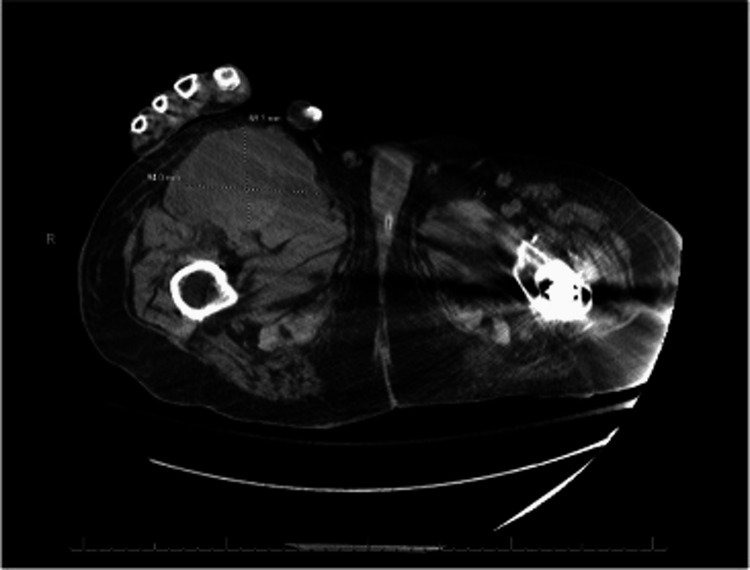
CT abdomen/pelvis without contrast A 4 mm right-sided non-obstructing nephrolith and additional bilateral punctate nephrolithiasis without evidence of hydroureteronephrosis was observed along with a large right anterior thigh fluid collection (6.9 x 1.4 cm) with the focus on internal hemorrhage corresponding to previously seen pseudoaneurysm. A large focal stool burden in the rectum, concerning impaction was obseved. A Foley catheter was in place and urinary bladder mural thickening was suspected.

**Figure 2:**
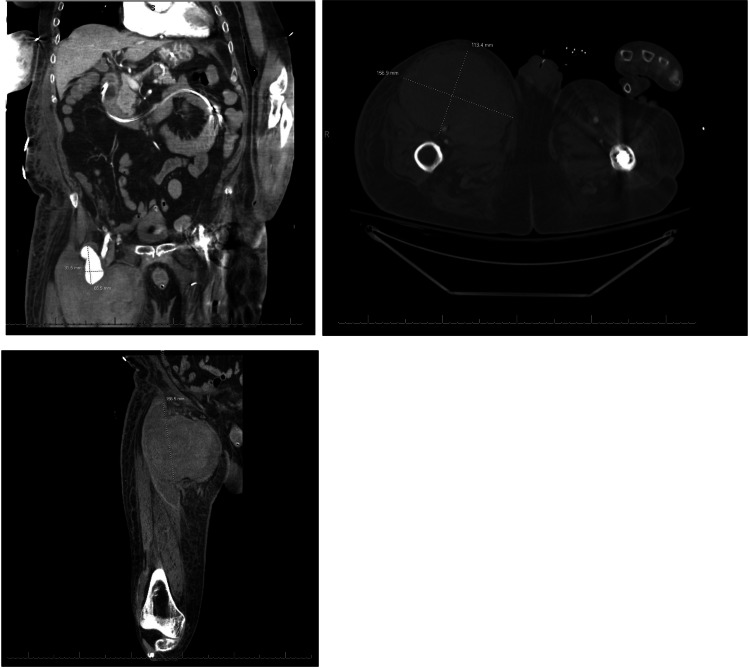
Follow-up CT abdomen/pelvis with contrast Interval increase in the size of the right proximal thigh CFA pseudoaneurysm now measuring 11.2 x 15.4 cm. Small bilateral pleural effusions with left lower lobe consolidation opacity. May represent a combination of atelectasis with the aspiration of infection.

Two weeks later, he presented to our internal medicine team. He was lethargic and oriented only to person and place. A systolic murmur was present at the left lower sternal border and the lungs were clear to auscultation. Lower extremities were significant for 1+ pitting edema bilaterally and a right femoral mass with mild tenderness to palpation. CT imaging of the abdomen/pelvis was significant for a 4 mm right-sided non-obstructing nephrolith and a large right anterior thigh fluid collection measuring 6.9 x 1.4 cm with internal hemorrhage corresponding to a previously seen pseudoaneurysm (Figure 1). He initially improved on antibiotics (sensitivity-directed carbapenem) but improvement stagnated after a few days and the size of the right femoral mass enlarged over this time period. Follow-up imaging over subsequent days (including CT and arterial duplex) showed progressive enlargement of the pseudoaneurysm and development of partial thrombosis and hematoma. The patient was transfused with 1 unit of RBC for acute anemia associated with hematoma formation. Shortly thereafter, the patient was transferred to the ICU for hypotension, tachypnea, atrial fibrillation with the rapid ventricular response, and lactic acidosis consistent with septic shock. Physical exam showed continued expansion of the right thigh mass in addition to erythematous skin changes and worsening tenderness to palpation. Given the overall picture, concern at that time was for the seeding of a pseudoaneurysm from bacteremia. Repeat CT imaging showed an interval increase in the size of the hematoma from 11 x 4.4 cm to 11.2 x 15.4 cm (Figure 2). The patient was taken up for surgical exploration of the groin with hematoma evacuation and stenting of the right common femoral artery, profunda femoris artery, and superior femoral artery. The operation was complicated by bradycardia followed by asystole requiring ACLS and Return Of Spontaneous Circulation (ROSC) was achieved after one round.

Tissue cultures obtained during surgery, as well as blood and urine cultures, were all positive for ESBL *Proteus mirabilis*. Infectious Diseases was consulted and the patient was restarted on ertapenem. Later he was placed on ervacycline. The post-surgical course was further complicated by severe wound necrosis requiring multiple surgical debridements and wound vac exchanges.

## Discussion

Bacterial causes of mycotic pseudoaneurysms mostly consist of *Staphylococcus aureus*, *Salmonella spp,* and *Pseudomonas aeruginosa*. The major bacterial organisms that can cause Mycotic pseudoaneurysms fall into the following categories: 55% belong to the Gram-positive cocci, with *S. aureus* accounting for 45% and streptococci accounting for 10% of the infections [1]. Additionally, *Salmonella* accounts for another 30-40% [2] leaving about 5-15% of pseudoaneurysms that are attributed to infection by a different bacteria. We had a unique case of a femoral pseudoaneurysm found to be caused by *Proteus mirabilis*. In our patient, the mycotic pseudoaneurysm was possibly seeded from a left heart catheterization procedure completed approximately six months prior to admission. However, it is unclear whether the initial source of *Proteus* infection stemmed from a urinary, cardiac, or vascular origin.

The treatment of mycotic pseudoaneurysms has been a heavily debated topic amongst infectious disease and vascular surgeons for many years. While there are currently no clinical trials on the management of mycotic pseudoaneurysms, the most common form of management of these mycotic pseudoaneurysms includes both antibiotic therapies along with surgical debridement, with or without revascularization [3]. Due to our patient having many comorbidities as outlined in the case report, a decision was made to keep the patient on antibiotics till he became asymptomatic. Our patient was placed on Ertapenem followed by Eravacycline with serial washouts and wound vacs to help heal the wound. Ertapenem was selected due to its very broad spectrum of activity that includes all anaerobes and many aerobic gram-positive and gram-negative organisms [4]. Eravacycline was used because of the lack of improvement from Ertapenem therapy and the patient had finished the recommended 14-day course [5,6]. Eravacycline has similar coverage as Ertapenem with broad-spectrum activity against Gram-positive cocci and Gram-negative *bacilli, *and it does not exhibit a loss of antibacterial activity against isolates expressing tetracycline ribosomal protection genes or most tetracycline efflux resistance genes [7,8]. The patient underwent four debridements and two wound vac changes throughout the course of admission as well as a hematoma evacuation. The patient was able to tolerate all procedures well and had improvement in clinical symptoms following each debridement for roughly 2-3 days. 

## Conclusions

This case shows the benefit of combined surgical intervention in cases of mycotic pseudoaneurysms. Although the patient improved significantly over his course in the hospital and was fit to be discharged from acute care, unfortunately, he passed away due to aspiration pneumonia within 90 days. In a patient with fewer comorbidities, earlier surgical intervention to debride the cavity of the necrotic or infected tissue could provide significant improvement of clinical symptoms and potentially have a curative effect in conjunction with antibiotics.
